# Expression of *Ciona intestinalis* Variable Region-Containing Chitin-Binding Proteins during Development of the Gastrointestinal Tract and Their Role in Host-Microbe Interactions

**DOI:** 10.1371/journal.pone.0094984

**Published:** 2014-05-02

**Authors:** Assunta Liberti, Daniela Melillo, Ivana Zucchetti, Lenina Natale, Larry J. Dishaw, Gary W. Litman, Rosaria De Santis, Maria Rosaria Pinto

**Affiliations:** 1 Department of Animal Physiology and Evolution, Stazione Zoologica Anton Dohrn, Villa Comunale, Napoli, Italy; 2 Department of Pediatrics, University of South Florida College of Medicine, St Petersburg, Florida, United States of America; Laboratoire Arago, France

## Abstract

Variable region-containing chitin-binding proteins (VCBPs) are secreted, immune-type molecules that have been described in both amphioxus, a cephalochordate, and sea squirt, *Ciona intestinalis*, a urochordate. In adult *Ciona*, *VCBP-A*, *-B* and *-C* are expressed in hemocytes and the cells of the gastrointestinal tract. VCBP-C binds bacteria in the stomach lumen and functions as an opsonin *in vitro*. In the present paper the expression of VCBPs has been characterized during development using *in situ* hybridization, immunohistochemical staining and quantitative polymerase chain reaction (qPCR) technologies. The expression of *VCBP-A* and *-C* is detected first in discrete areas of larva endoderm and becomes progressively localized during differentiation in the stomach and intestine, marking the development of gut tracts. In “small adults” (1–2 cm juveniles) expression of *VCBP-C* persists and *VCBP-A* gradually diminishes, ultimately replaced by expression of *VCBP-B*. The expression of *VCBP-A* and *-C* in stage 7–8 juveniles, at which point animals have already started feeding, is influenced significantly by challenge with either Gram-positive or -negative bacteria. A potential role for VCBPs in gut-microbiota interactions and homeostasis is indicated.

## Introduction

The variable region containing chitin-binding proteins (VCBPs), which possess a domain organization consisting of a leader peptide, two tandem N-terminal immunoglobulin V-type domains and a single C-terminal chitin-binding domain, belong to a multigene family encoding secreted proteins [1], [2]. The VCBP molecules were identified first in the cephalochordate *Branchiostoma floridae* [1]. In this species, VCBPs have been characterized extensively at the genetic and structural levels [1], [3]–[5]. Expression patterns, genetic diversity and structural similarities with V-type domains of immunoglobulins and T cell receptors suggest that VCBPs represent a unique gut-associated form of innate immune proteins [1] and could reflect an important transition between non-rearranging immune molecules and the conventional rearranging antigen-binding receptors of jawed vertebrates [3].

Three canonical *VCBP* genes, *VCBP-A*, *-B* and *-C*, have been identified in *Ciona intestinalis*, a urochordate [2], [3], [6]. In the adult organism, these genes exhibit specific patterns of expression in different immune-competent tissues. *VCBP-B* and *-C* are expressed in some of the cell types present in the stomach epithelium, *VCBP-C* transcripts have been detected in the intestine [2] and *VCBP-A* and *-C* are expressed in the granular amoebocytes that populate the connective tissue surrounding the stomach (*lamina propria*) as well as in the circulating hemocytes [2].

Functional studies have revealed that VCBP-C acts as a secreted immune-type molecule, capable of binding the surfaces of both Gram-positive and -negative bacteria in the stomach lumen. VCBP-C functions as an opsonin in an *in vitro* assay by increasing the rate of phagocytic activity of granular amoebocytes. Phagocytic activity is attributable largely to the V-type domains, thereby establishing a role for V region-containing molecules in immune recognition at an early point in phylogeny [2]. It is most likely that VCBPs function is confined largely to the gut where these secreted molecules may be integral components of gut homeostasis [2], [7]. VCBPs represent a particularly informative example of how alternative mechanisms of immune-type function have become uniquely adapted to the physiology of different species [8].

The low complexity of the *Ciona* gut coupled with its striking anatomical and cellular analogies to the human gut [2], [9], as well as a growing awareness of the broad physiological significance of microbiota that colonize the gut of healthy organisms, underscore the value of this species as a developmental model. Mapping of VCBP expression during development and metamorphosis has been carried out, with the primary focus directed to the stages relating to feeding initiation when the internal compartments of the body first come in contact with the microbial environment. The expression patterns of VCBP genes serve as a particularly informative marker of gut tract differentiation. The finding that VCBPs expression is influenced by the introduction of Gram-positive or -negative bacteria at the 7–8 juvenile stage, underscores a potential role for VCBPs in gut homeostasis at an early stage of gut-microbiota interactions.

## Results

### *VCBPs* are differentially expressed during development

#### VCBP qPCR analysis during embryo development

**Figure 1:**
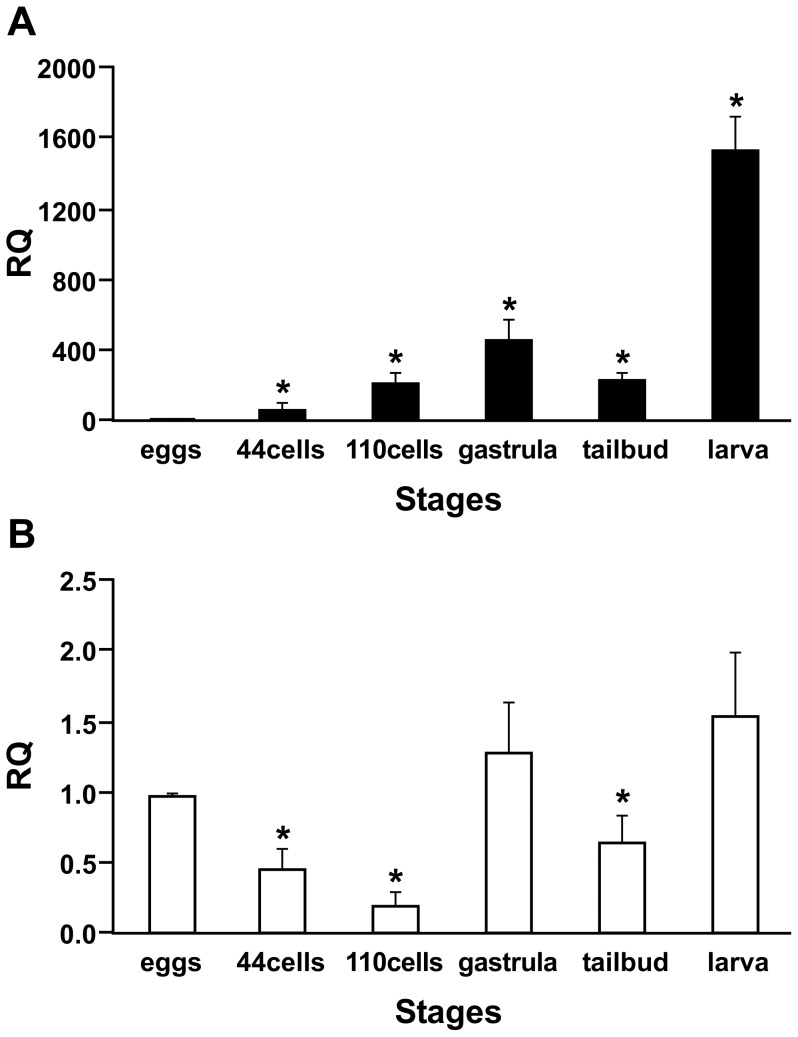
Expression levels of *VCBP-A* and *VCBP-C* during *Ciona* embryo development determined by qPCR. The graphs indicate the relative quantity (RQ) of *VCBP-A* (A) and *VCBP-C* (B) transcripts compared to unfertilized eggs. All data were normalized against cytoskeletal actin mRNA levels. The results are presented as the mean value ΔΔCt and SD of three independent experiments, performed in triplicate. Asterisk indicates p value<0.05.

Only VCBP-A and -C transcripts could be detected in qPCR carried out on samples of unfertilized eggs and developmental stages prior to the larval stage. At the larval stage (Fig. 1A), the expression of *VCBP-A* increases significantly, whereas the expression of *VCBP-C* is not increased significantly compared to the earlier developmental stages (Fig. 1B).

#### VCBP expression from 44 cells stage to larva

**Figure 2:**
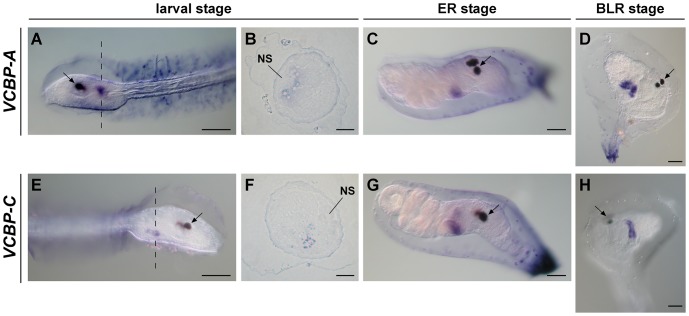
WISH of *VCBP-A* and *-C* at larval stage and at early stages of metamorphosis. In the early-middle swimming larva, *VCBP-A* (A) and *VCBP-C* (E) are expressed in the dorsal endodermal cells and in the posterior lateral endoderm, respectively. Cross sections cut at the level of the dotted lines confirm these expression patterns (B and F). At the ER stage *VCBP-A* (C) and *VCBP-C* (G) are localized in the intestine disc, with an overlapping pattern. At the BLR stage, *VCBP-A* (D) is expressed in the primordium of the stomach, and *VCBP-C* (H) in the primordium of the intestine. NS, nervous system; arrow, sensory organs. (Scale bars: A, C-E, G, H, 50 µm; B and F, 15 µm).

*In situ* hybridization (ISH) with *VCBP-A*, *-B* and *-C* riboprobes does not detect transcripts in defined territories at the 44-cell, 110-cell, gastrula and tailbud stages (data not shown). At the early-middle larval stage (free swimming larva) (stage 27–28, FABA2 developmental staging, http://chordate.bpni.bio.keio.ac.jp/faba2/2.2/top.html), *VCBP-A* mRNA is expressed in some dorsal endodermal cells lining the area corresponding to the neck of the nervous system (Fig. 2A and B); slight variations in both extent and intensity are seen, depending on the specimen examined. By contrast, *VCBP-C* is expressed in the posterior lateral region of endoderm facing the mesenchyme pouches (Fig. 2E and F). The expression patterns can be interpreted further in semi-thin transverse sections in which *VCBP-A* expression is localized in endodermal cells at the border of the larval nervous system (Fig. 2B), whereas *VCBP-C* expression is confined to a few more ventrally located cells (Fig. 2F).

Based on the fate map of the territories identified in *H. roretzi* larva [10], hybridization of the *VCBP-A* and *-C* probes is localized to the prospective regions that give rise to the oesophagus, stomach and the intestine in the adult. *VCBP-A* expression is localized in the presumptive territory of the stomach and the oesophagus, whereas *VCBP-C* is expressed in the presumptive territory of the intestine. A schematic representation of *VCBP* genes expression from the larva through stage 8 of 2^nd^ ascidian juvenile is depicted in Figure S1. Corresponding controls performed with sense probes are shown in Figure S2 (A and B).

#### Early and beginning of late rotation stage

At the early rotation (ER) stage, the expression patterns of *VCBP-A* and *-C* are overlapping and restricted to the structure identified as the intestine disc [11], located in the ventral part of the organism (Fig. 2C and G). Later in metamorphosis, at the beginning of the late rotation (BLR) stage, *VCBP-A* and *-C* again exhibit defined expression patterns in two different regions of the same structure. *VCBP-A* is expressed as a round mass of cells corresponding to the stomach primordium (Fig. 2D), whereas *VCBP-C* expression is localized to a tube-shaped structure that corresponds to the developing intestine (Fig. 2H). Figure S2 (C-F) shows the controls performed with the corresponding sense probes.

#### Stage 4 of 1^st^ ascidian juvenile

**Figure 3:**
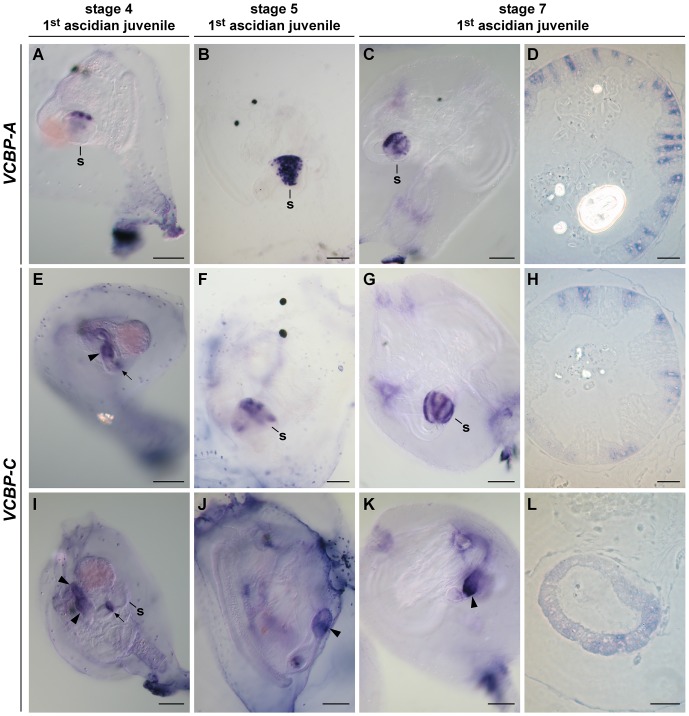
WISH of *VCBP-A* and *-C* at stages 4, 5 and 7 of 1^st^ ascidian juvenile. *VCBP-A* is localized in the stomach structure in all the stages examined (A–C). In particular, semi-thin sections (D) of WISH samples at the stage 7 indicate that *VCBP-A* is expressed in scattered cells of the stomach epithelium. *VCBP-C* is localized in part of the developing intestine and in a ring-shaped structure at the junction between the stomach and the intestine (E and I) at stage 4, whereas at stage 5 and 7, it is expressed in both the stomach (F and G) and in the intestine (J and K). Semi-thin sections of WISH samples confirm that at stage 7 *VCBP-C* is confined to groups of cells localized in the ridges of the stomach (H) and distributed evenly in the intestine (L). s, stomach; arrow, ring-shaped structure; arrowhead, intestine. (Scale bars: A, B, E, F, I and J, 50 µm; C, G and K, 100 µm; D, H and L, 15 µm).

**Figure 4:**
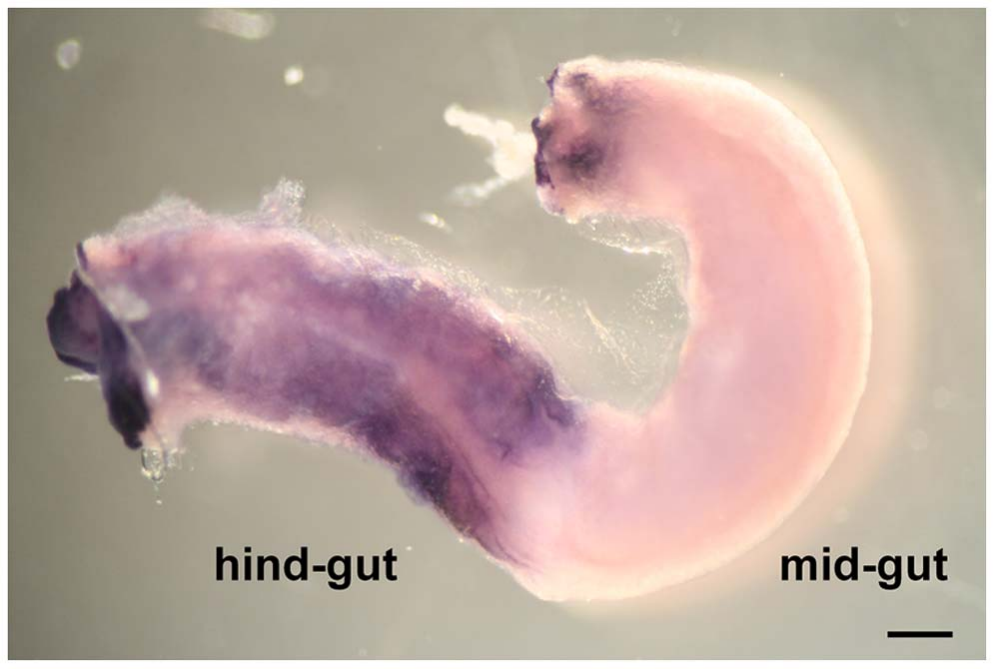
Expression pattern of *VCBP-C* in the intestine of the adult as determined by WISH. *VCBP-C* expression is localized in the first tract of the mid-gut and in the hind-gut. (Scale bar: 600 µm).

Gut differentiation proceeds, although feeding has not commenced, at stage 4 in the early development of juvenile ascidians [12]; the oesophagus, stomach and intestine can be distinguished. *VCBP-A* mRNA is localized in the stomach and restricted only to some cells in the region closer to the oesophagus (Fig. 3A). By contrast, *VCBP-C* is expressed in a small ring of cells at the border between the stomach and the intestine, as well as in a more extended region of the intestine that corresponds to the hind-gut (Fig. 3E and I). It has not been possible to detect expression of *VCBP-C* in the mid-gut. In fully differentiated adult intestine, whole mount *in situ* hybridization (WISH) reveals expression of *VCBP-C* in a ring-shaped structure at the junction between the stomach and the intestine, and in the hind-gut; as in stage 4 of 1^st^ ascidian juvenile, the mid-gut is clear (Fig. 4). The corresponding controls carried out with sense probes are shown in Figure S2 (G and H).

#### Stage 5 of 1^st^ ascidian juvenile

At stage 5 of 1^st^ ascidian juvenile, after the onset of feeding, *VCBP-A* is expressed in an increasing number of scattered cells in the stomach (Fig. 3B). Similarly, *VCBP-C* expression is maintained within the intestine and extends further to a few cells in the stomach (Fig. 3F and J). This pattern of expression differs markedly from that exhibited by *VCBP-A*. Controls are shown in Figure S2 (I and J).

#### Stage 7 of 1^st^ ascidian juvenile

At stage 7 of 1^st^ ascidian juvenile, differentiation of the digestive tract permits a more detailed interpretation of the expression patterns. *VCBP-A* mRNA is distributed evenly in scattered cells of the stomach epithelium (Fig. 3C) and *VCBP-C* continues to be expressed in both the stomach and the intestine, primarily in the proximal region of the hindgut (Fig. 3G and K). In the stomach, *VCBP-C* exhibits a distinctive striped pattern of hybridization (Fig. 3G).

**Figure 5:**
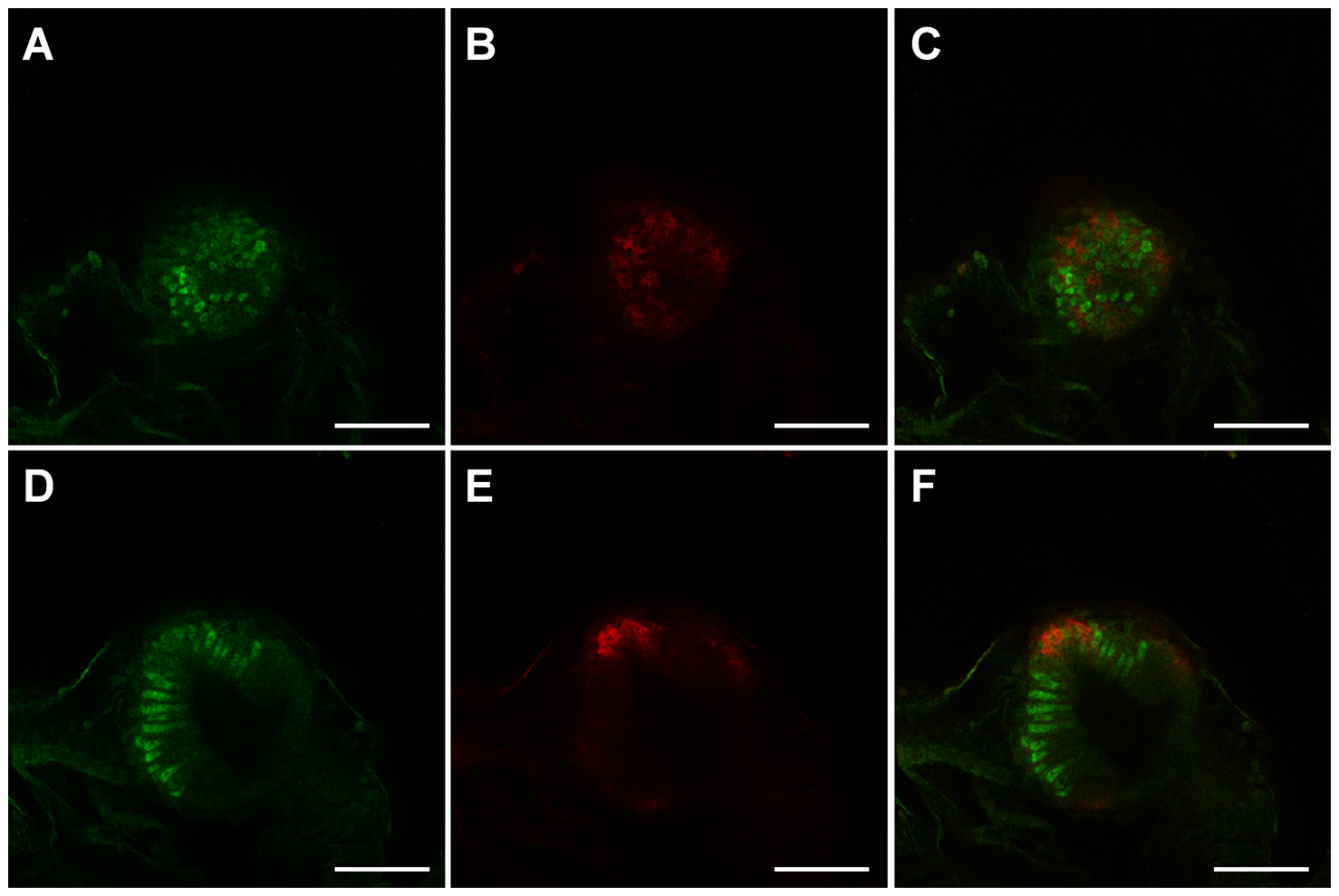
Double staining WISH pattern of *VCBP-A* and *-C* at stage 7 of 1^st^ ascidian juvenile. *VCBP-A* (A and D) and *VCBP-C* (B and E) are expressed in different cells of the stomach epithelium, as observed in the merged confocal microscopy images (C and F). A–C and D–F represent images acquired at two different planes of the same z-stack. (Scale bar: 50 µm).

Additional details can be discerned from WISH of semi-thin sections of the stomach and intestine using *VCBP-A* and *-C* probes (Fig. 3D, H and L). *VCBP-A* and *-C* are not expressed in all cells of the stomach (Fig. 3D and H). Specifically, *VCBP-A* is detected primarily in scattered cells in the stomach wall (Fig. 3D), whereas expression of *VCBP-C* is confined to groups of cells that line the ridges of the stomach (Fig. 3H). A similar pattern was observed in whole mount preparations, although the epithelium at this stage exhibits only a slightly waved characteristic and lacks the folding seen in the adult. *VCBP-C* shows a uniform pattern of expression in the intestine (Fig 3L). ISH performed with the sense probes is shown in Figure S2 (K and L). Double staining ISH using both *VCBP-A* and *-C* probes indicates that *VCBP-A* and *-C* are expressed in different regions and in different cells of the stomach epithelium (Fig. 5A–F); co-localization of signals was not detected.

#### Stage 8 of 2^nd^ ascidian juvenile

**Figure 6:**
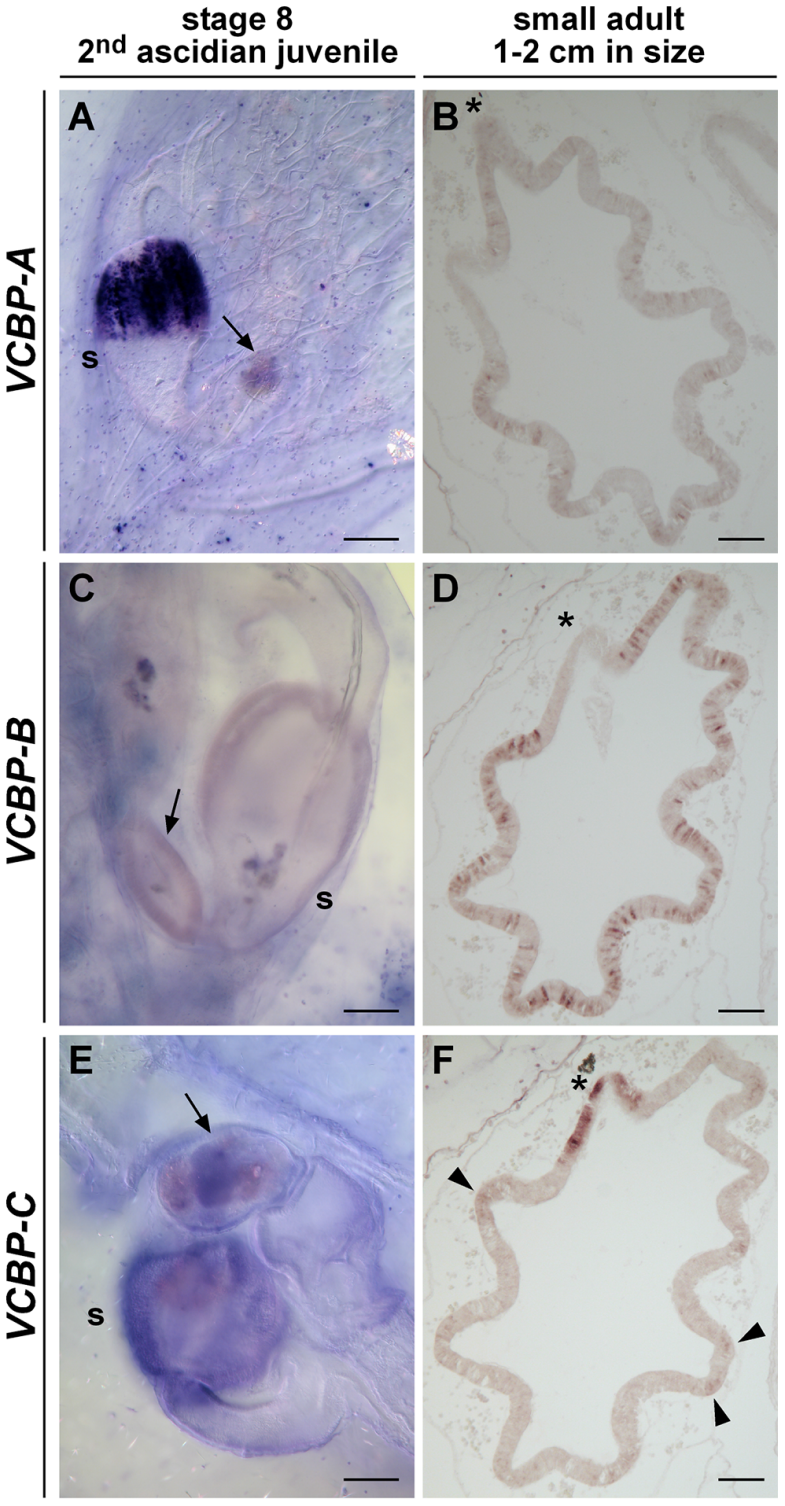
WISH of *VCBPs* at stage 8 of 2^nd^ ascidian juvenile and on ‘small adult’ sections. At stage 8, *VCBP-A* (A) is still expressed in the stomach cells, *VCBP-B* (C) is not yet expressed and *VCBP-C* (E) expression is localized in both the stomach and the intestine. ISH on stomach sections, including the region of the junction between the stomach and the intestine (asterisk) of “small adults”, shows that *VCBP-A* (B) and *VCBP-B* (D) have faint and strong expression patterns, respectively. *VCBP-C*, instead, is expressed in the crypts of the stomach ridges (arrowheads) and in the area at the junction between the stomach and the intestine. s, stomach; arrow, intestine. (Scale bars, 50 µm).

At stage 8 of 2^nd^ ascidian juvenile, the last juvenile stage examined, the hybridization patterns of *VCBP-A* and *-C* are equivalent to those seen in stages 5 and 7 of the 1^st^ ascidian juvenile, i.e. *VCBP-A* RNA is detected only in the stomach (Fig. 6A), whereas *VCBP-C* RNA is detected in both the stomach and the intestine (Fig. 6E). Under identical conditions of hybridization, it was not possible to detect expression of *VCBP-B* RNA (Fig. 6C). Corresponding controls performed with sense probes are shown in Figure S2 (M and N).

#### VCBPs expression on stomach sections of “small adults”

Animals older than stage 8 of 2^nd^ ascidian juvenile are approximately 1–2 cm in size, resemble the adult form morphologically and are referred to here as “small adults”. ISH carried out on stomach sections of these individuals, reveals that *VCBP-A* is expressed more weakly compared to that seen in earlier developmental stages (Fig. 6B). *VCBP-B* expression is detected first in scattered cells of the stomach epithelium (Fig. 6D) and follows the same expression pattern that is observed in the adult [2]. Similarly, *VCBP-C* expression retraces the same pattern of hybridization observed in the 7 and 8 juvenile stages, as seen in adult stomach, particularly in the crypts of the stomach ridges (Fig. 6F). The expression of *VCBP-C*, but not *VCBP-B*, is particularly conspicuous in the junction between stomach and intestine (Fig. 6B, D and F). Figure S2 (O-Q) represents the corresponding controls.

### VCBP proteins are detected at BLR stage and stage 7 of 1^st^ ascidian juvenile

**Figure 7:**
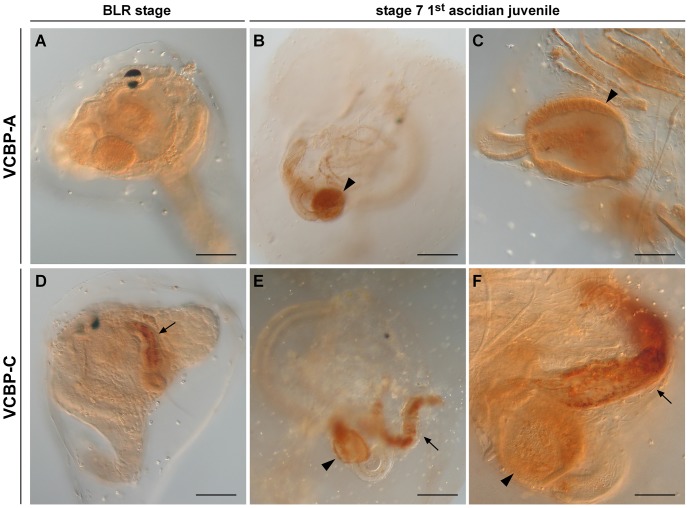
VCBP-A and -C localization at BLR stage and stage 7 of 1^st^ ascidian juvenile by WIHC. Whereas VCBP-A (A) does not show a defined localization at the BLR stage, VCBP-C protein (D) is localized in a tube-shaped structure corresponding to the developing intestine. At stage 7, VCBP-A is expressed in the stomach cells (B and C) and VCBP-C (E and F) is expressed in the stomach and intestine. Arrow, intestine; arrowhead, stomach. (Scale bars: A, B, D and E, 50 µm; C and F, 100 µm).

Whole mount immunohistochemistry (WIHC) employing specific anti-VCBP-A and -C antibodies has been carried out on BLR stage and stage 7 of 1^st^ ascidian juveniles. VCBP-A protein is not detected in the stomach at the BLR stage (Fig. 7A) and VCBP-C is localized to the developing intestine (Fig. 7D). At stage 7 of 1^st^ ascidian juvenile, VCBP-A protein is detected in the stomach (Fig. 7B and C) whereas VCBP-C protein is detected in both the stomach and the intestine (Fig. 7E and F). Overall, the results of specific antibody staining are consistent with the expression patterns observed for *VCBP-C* mRNAs, i.e. VCBP-C is expressed throughout the BLR stage and stage 7 of 1^st^ ascidian juvenile developmental stages. Whereas VCBP-A mRNAs can be detected from BLR stage to stage 7 of 1^st^ ascidian juvenile, VCBP-A protein is detected only in the stage 7 of 1^st^ ascidian juvenile. Figure S3 shows the controls performed with the corresponding pre-immune sera.

### VCBP proteins are present in stomach tissues

Previous IHC screening [2] using antibodies specific for VCBP-C indicates that its distribution is confined to scattered cells of the stomach epithelium and granular amoebocytes of the *lamina propria*. In order to further localize VCBPs in the gastrointestinal tract, immunohistochemical analyses were carried out on sections of adult stomach employing an anti-VCBP-A/B (that recognizes epitopes shared by both VCBP-A and -B), an anti-VCBP-A, and an anti VCBP-C antibody.

**Figure 8:**
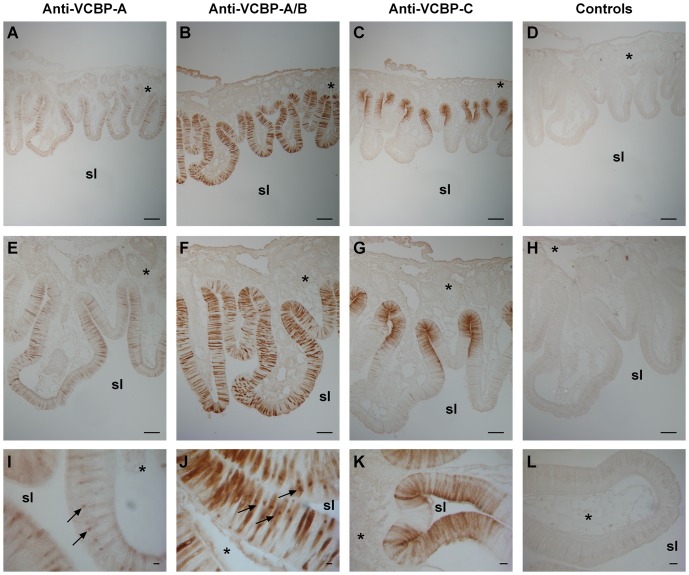
Distribution of VCBP-A, -B and -C in adult stomach determined by immunohistochemistry. VCBP-A is identified at the apical portions of the cells located in the crypts, in the basal parts of the cells located in the apical area of the ridges (A, E) and in large vacuoles of scattered epithelial cells (I). Anti-VCBP-A/B antibody shows an expression pattern in cells scattered throughout the stomach epithelium (B, F and J). VCBP-C is detected in cell types that reside in the crypts of the stomach epithelium (C, G and K). Control experiments with preimmune rabbit IgG (D, H and L). sl, stomach lumen; asterisk, *lamina propria*. (Scale bars: A–D, 50 µm; E–H, 100 µm; I and J, 5 µm; K and L, 10 µm).

Although *VCBP-A* transcripts were not detected in the stomach cells, IHC indicates that VCBP-A protein is localized in the apical part of the cells in the crypts, and in the basal part of the cells forming the tip of the ridges (Fig. 8A and E). Moreover, the protein also is present in large vacuoles within scattered epithelial cells (Fig. 8I). Similar localization in the vacuoles also is observed in sections immunostained with the anti-VCBP-A/B antibody, which also identifies the VCBP-B protein (Fig. 8J). VCBP-B is localized in cells scattered throughout the stomach epithelium that either may correspond to different cell types or represent various differentiation stages of the same cell type (Fig. 8B, F and J). VCBP-C is localized in the cell types that reside at the bases of the crypts of the numerous ridges that form the stomach structure (Fig. 8C, G and K). Controls carried out with IgG purified from the corresponding pre-immune sera are shown in Figure 8 (D, H and L).

**Figure 9:**
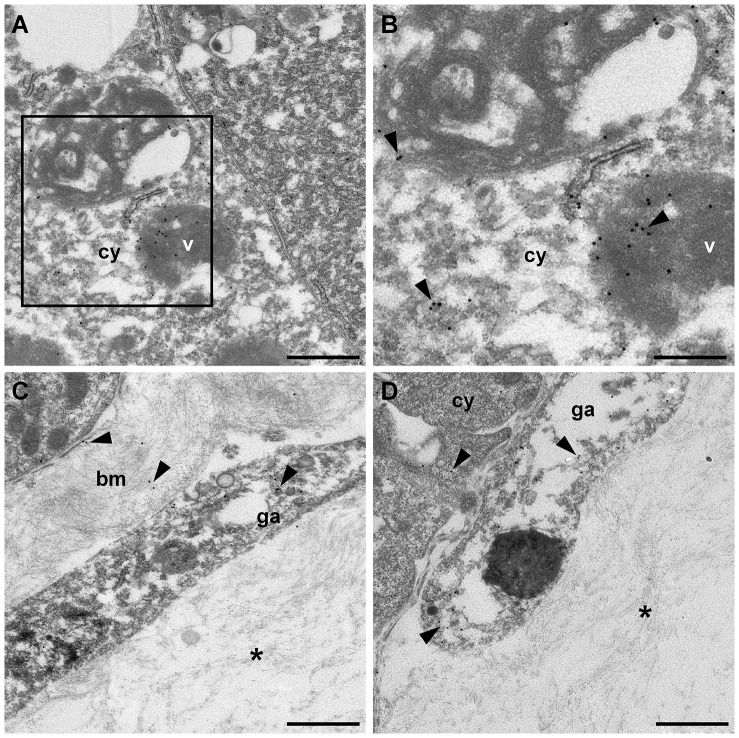
Immunogold localization of VCBP-A by anti-VCBP-A antibody in adult stomach sections. In stomach epithelium VCBP-A is detected in both the cytoplasm and within large vacuoles of the absorptive cells (A and B). In the *lamina propria*, gold particles are localized in the granular amoebocytes (C and D). In some instance, amoebocytes expressing VCBP-A are found associated with the basement membrane of the epithelium (C) and apposed to the plasma membrane of the epithelial cells (D); gold particles also are present in the basal rim of the plasma membrane of the stomach epithelium (D). (B) Higher magnification of the area marked in (A). bm, basement membrane; cy, cytoplasm; ga, granular amoebocyte; v, vacuole; arrowheads, gold particles; asterisk, *lamina propria*. (Scale bars: A, C and D, 1 µm; B, 0,5 µm).

In order to clarify why VCBP-A protein could be detected in an as yet unidentified stomach cell, in which *VCBP-A* transcripts were not detected by ISH, IHC analysis was carried out at the ultrastructural level. VCBP-A protein can be localized to the cytoplasm and within the large vacuoles of the absorptive cells (Fig. 9A and B). VCBP-A also could be detected in the *lamina propria*, both as a secreted molecule and in the granular amoebocytes populating this compartment (Fig. 9C). Quite often this type of hemocyte is associated with the basement membrane of the stomach cells and apposed to their plasma membrane (Fig. 9C and D). In these observation fields, VCBP-A expression also is detected in the basal rim of the plasma membrane and in the adjacent cytoplasmic areas of the epithelial cells (Fig. 9C and D).

### Administration of bacteria affects VCBPs expression

**Figure 10:**
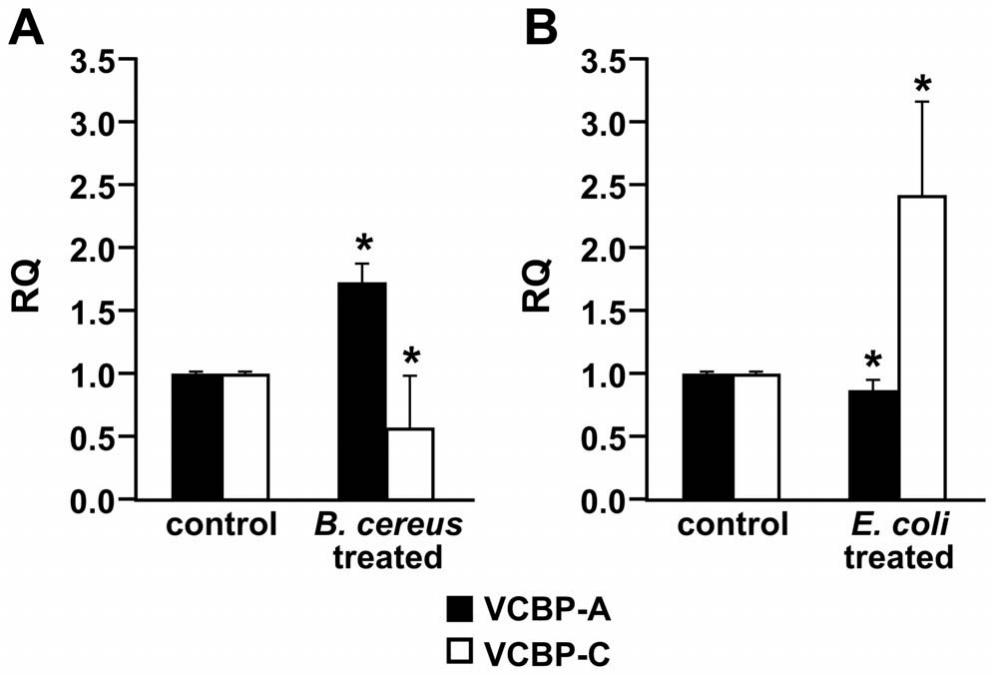
qPCR of *VCBP-A* and *-C* at stage 7-8 of ascidian juvenile following bacteria treatment. Three hours after *B. cereus* treatment, qPCR experiments reveal a significant increase in the relative quantity (RQ) of *VCBP-A* transcripts and a significant decrease in *VCBP-C* mRNA (A). After treatment with *E. coli*, a significant two-fold increase of *VCBP-C* expression is noted in the histogram (B). The results are presented as the mean value of ΔΔCt of each group compared to untreated stage 7 of 1^st^ ascidian juvenile. All data were normalized against cytoskeletal actin mRNA levels. The results are presented as the mean value ΔΔCt and SD of three independent experiments. Asterisk indicates p value<0.05.

Stage 7-8 juveniles were allowed to ingest either Gram-positive *Bacillus cereus* or Gram-negative *Escherichia coli* in order to determine the influence of bacteria challenges on the expression of VCBP-A and -C. Using total RNA in a qPCR analysis, a significant increase in the expression of *VCBP-A*, with a concomitant decrease in expression of *VCBP-C*, was observed with the *B. cereus* challenge (Fig. 10A). The opposite result occurred in response to the *E. coli* challenge in which a minor decrease in the expression of *VCBP-A* mRNA and a two-fold increase in the expression of *VCBP-C* mRNA (Fig. 10B) were observed.

## Discussion

Although it has been shown that the gut is the major site of expression of VCBPs, it was unclear when these genes are expressed in the developing animal. It now is apparent that localized expression of VCBPs by ISH cannot be detected until the tailbud stage. In *H. roretzi*, blastomeres already committed to give rise to the digestive organs have been identified at the 110-cell stage [10], [12], [13]. In *Ciona*, none of the blastomeres at this stage exhibits expression of VCBP genes, likely due to detection/sensitivity limits.

qPCR analysis performed on 44-cells and 110-cells embryos detects very low expression levels of only *VCBP-A* and *-C*, thus confirming the ISH results. The expression of both these *VCBP* genes in gastrula and tailbud are markedly lower than that observed in larvae. Taken together, these observations indicate that *VCBP-A* and *-C* are not expressed abundantly in those territories fated to give rise to the gut.

*VCBP* genes exhibit complex expression patterns through subsequent stages of development. *VCBP-A* can be detected in the presumptive territories of the digestive tract from the larva stage through the “small adult” stage, whereas *VCBP-B* only can be detected in the stomach starting from the “small adult” stage and exhibits the same pattern of expression as *VCBP*-A, in scattered cells of the stomach epithelium. *VCBP-A* may function during the differentiation of the digestive tract, whereas the role of *VCBP-B*, which shares a high degree of sequence similarity with *VCBP-A*, may be restricted to the adult. At present it is not possible to further classify the actual cell types that express *VCBP-A* and whether or not they correspond to the cells expressing *VCBP-B*.

Comparison of the expression patterns of *VCBP-A* and *-C* from the larval stage throughout metamorphosis to adulthood sheds new light on the pathways of differentiation of the gastrointestinal tract. Based on the fate map of the territories identified in *H. roretzi* [10], *VCBP-A* and *-C* expression at the larval stage is localized in two distinct territories of the endoderm that correspond to the presumptive regions that give rise to the stomach and the oesophagus (*VCBP-A*), and the intestine (*VCBP-C*). A recent report, which characterizes the territories giving rise to the digestive tract in *Ciona*, confirms these observations [14]. Specifically, folding and closure of the endodermal epithelia in the central-to-right posterior trunk form the tubular structure of the oesophagus and stomach. In the left posterior trunk, the accumulation and rearrangement of the cells that originate from the endodermal strand, a row of cells localized in the tail under the notochord, form the intestine [14]. *VCBP-A* expressing cells that participate in the formation of the stomach are localized in the central-to-right posterior trunk of the larva endoderm, as seen on semi-thin sections of *VCBP-A* WISH. Conversely, *VCBP-C* is expressed in cells that produce part of the intestine and do not correspond to those migrating from the endodermal strand. *VCBP-C* is expressed in the cells of the ventral posterior trunk in the early-middle swimming larva stage when the cells of the endodermal strand have yet to initiate their migration, which begins at the early adhesion stage [14]. It appears as if *VCBP-C* expression marks those cells committed to give rise to the part of the intestine that does not derive from the endodermal strand. The results presented here also are consistent with recent data regarding the formation of the digestive tract throughout the rotation stage [14]. Although it was not the aim of these investigations, the differential expression of *VCBP-A* and *-C* during metamorphosis not only allows us to follow the development of the digestive tract, the morphogenesis of which largely is unknown, but also to trace the formation of distinctive areas of the stomach and intestine in early phases of organ differentiation. A direct role of VCBPs in gut differentiation cannot be ruled out.

By monitoring *VCBP-A* and *-C* expression during metamorphosis, it has been found that both these genes are expressed in the intestine disc at the ER stage; however, regionalized expression cannot be discerned. From the BLR stage until the end of metamorphosis, *VCBP-A* and *-C* exhibit markedly different patterns of expression. Although localization of *VCBP-A* is constant in the developing stomach throughout metamorphosis, *VCBP-A* transcripts are not present in the entire structure but rather are confined to scattered cells of the epithelium, localized near the oesophagus, particularly in the early phase of metamorphosis. By contrast, expression of *VCBP-C* at early metamorphosis (BLR stage and stage 4 of 1^st^ ascidian juvenile) is localized in distinctive tracts in the developing intestine. At stage 4 of 1^st^ ascidian juvenile, *VCBP-C* expression is localized to a small ring of cells at the border between the stomach and the intestine, as well as to a region of the hindgut. This pattern of expression also occurs in adults, as revealed by WISH on adult intestine, thus indicating that the regionalization of the intestine is determined in the early phases of organ differentiation. Collectively, the results demonstrate the clear partitioning of VCBP expression into distinct regions of the gut as well as dynamic changes in the patterns of expression as gut development proceeds.

From stage 5 of 1^st^ ascidian, at the onset of feeding, *VCBP-C* expression can be detected in the stomach. Previously it was shown that *VCBP-C* interacts with bacteria [2] and its expression in the stomach epithelium may correlate with the onset of interactions of the digestive system with the external environment. It is possible to assume that *VCBP-C*, perhaps in concert with *VCBP-A*, may play a role in establishing the gut microbiota that colonizes the digestive tract.

*VCBP-A* is expressed in cells scattered in the epithelium, whereas *VCBP-C* is localized in groups of cells at the base of the ridges typical of the adult stomach, but not the grooves and ridges, as demonstrated by double staining ISH with stage 7 of 1^st^ ascidian juvenile. The expression patterns of *VCBP* genes in distinct regions of the organ and thereby in different cell types is consistent with the functional divergence of various types of stomach cells but also may reflect different stages of differentiation of the same cell type.

These conclusions are validated further by the different timing of the expression of VCBP-A and -C proteins. Whereas *VCBP-C* mRNA is translated from BLR stage onwards, VCBP-A protein only can be detected at the stage 7 of 1^st^ ascidian juvenile. This finding, together with previous results indicating that VCBP molecules are secreted into the stomach lumen where they bind the bacterial surfaces and function as opsonins [2], prompted us to investigate whether or not the expression of VCBPs at stage 7-8 of ascidian juvenile is influenced by ingestion of different types of bacteria. The finding of an opposite response to the expression of *VCBP-A* and *-C* following exposure to either Gram-positive *B. cereus* or Gram-negative *E. coli* suggests the possibility that the VCBPs may serve a role(s) at the onset of gut colonization by microbiota.

The presence of V-type immunoglobulin domains, expression in the gut and ability to bind bacteria [2] led us previously to speculate that VCBPs exhibit broad similarities to vertebrate IgA, the class of mucosal immunoglobulins produced in mammals [15] as well as in equivalent forms seen in other vertebrates [16], [17]. IgA antibodies function mainly in a high-affinity mode to protect intestinal mucosal surfaces against colonization and invasion by pathogenic microbes. IgA also functions in a low-affinity mode to confine the dense commensal microbiota within the intestinal lumen through a process known as ‘immune exclusion’ [15]. IgA also can mediate apical-to-basolateral transcytosis of antigens across microfold cells, epithelial-like cells specialized in antigen-capture [18]. Based on these observations, a role for IgA in gut homeostasis has been proposed. In *Ciona* VCBPs may function in gut homeostasis in an analogous manner.

The findings that *VCBP-A* mRNA has not been detected in the stomach epithelium [2] but is detected in the granular amoebocytes of the *lamina propria*, where expression of the VCBP-A protein has been documented, suggest a non-endogenous origin of VCBP-A of which hemocytes seem to be the most likely source. Ultrastructural observations indicate that VCBP-A protein is released by the granular amoebocytes in the proximity of the plasma membrane of stomach cells facing the *lamina propria* and also is found in the basal area of the cytoplasm of absorptive cells, but never in the stomach lumen. VCBP-A protein is produced by the hemocytes and may be transferred through an unknown mechanism to the stomach absorptive cells where it accumulates in the large vacuoles. VCBP-A may have a role in the control of bacteria that have managed to penetrate the epithelial cells and/or to reach the *lamina propria*. It is possible that VCBP-B and -C could function in a process akin to immune exclusion. Irrespective of the functional roles of VCBPs, it is possible from their expression early in the formation of the digestive tract that they are integral to the overall development of the gut, the physiology of which now is recognized to be intimately involved in interactions with microbiota. The results of the expression analysis point to VCBPs as early and highly informative morphological markers of the digestive tract epithelium during development and differentiation.

## Experimental Procedures

### Ethics statement

The research described herein was performed on *Ciona intestinalis*, a marine invertebrate collected in the Gulf of Napoli (Italy), in locations that are not privately-owned nor protected in any way, according to the authorization of Marina Mercantile (DPR 1639/68, 09/19/1980, confirmed on 01/10/2000). The study did not involve endangered or protected species, and was carried out in strict accordance with European (Directive 2010/63) and Italian (Decreto Legislativo n. 116/1992) legislation for the care and use of animals for scientific purposes.

### Animal handling, fertilization and tissue preparation

For *in vitro* fertilization, eggs and spermatozoa were collected surgically from the gonoducts of animals exposed to constant light in order to induce gamete maturation. Embryos were raised in Millipore-filtered seawater at 18–20°C and samples at various stages were fixed for WISH or, after collection by low speed centrifugation, were frozen in liquid nitrogen for RNA extraction. Only the batches containing at least 90% of normally developed embryos were used. Embryo batches were allowed to develop in Petri dishes in circulating seawater under standard conditions until the juvenile stages. When the appropriate stages were reached, the animals were detached from the dishes and fixed for ISH and IHC, both performed either on sections or in whole mount. Stomach and intestine were surgically dissected from adult animals and appropriately fixed and processed for WISH and IHC experiments, for both electron and light microscopy.

### RNA isolation and cDNA synthesis

Total RNA was extracted from stomach, hemocytes, embryos at different developmental stages and juvenile samples using the SV Total RNA Isolation System Kit (Promega). Oligo(dT)-primed single-stranded cDNA was synthesized from the total isolated RNA using the SuperScript III First-Strand Synthesis System for RT-PCR (Invitrogen).

### *VCBP* riboprobe preparation

*VCBP*s riboprobes were prepared as previously described [2] using *VCBP*-specific primers, which produced single-band amplicons of the expected size: 463 bp for *VCBP-A/B* (sense primer, 5′-GGTACGAAGGACCTTTCCATC-3′; antisense primer, 5′-GTCCCGCATGGTGGCGCTAC-3′) and 714 bp for *VCBP-C* (sense primer, 5′-ATGTTTTGTGTATGTTGGTCTTATTG-3′; antisense primer, 5′-GACGCCTTTAGACCAGTTATCA-3′). The amplification products were cloned into pCRII-TOPO (Invitrogen) and verified by DNA sequencing. Transcription of antisense and sense RNA probes was carried out with a digoxigenin (DIG) RNA labeling kit (Roche) according to the manufacturer's instructions. *VCBP-A* and *-C* riboprobes to be used in the double staining WISH were labelled with Fluorescein-12-UTP and DIG-UTP, respectively, following the same procedure as for DIG-labelled riboprobes.

### Anti-VCBP antibodies

The anti-VCBPs antibodies, prepared as previously described [2] (AnaSpec, Fremont, CA), were against three synthetic peptides, TWYEGPFHLPDSENFT, TNDQHTEYDMVYTRTS, and KIGTFDLNSQQVEMEAGFTEMFID, corresponding to predicted immunogenic epitopes of the second IgV domain of VCBP-A/B, -C, and -A, respectively. The three anti-peptide specific antibodies were purified from rabbit immune serum by affinity chromatography using the corresponding synthetic peptides coupled to cyanogen bromide-activated Sepharose 4B (GE Healthcare Life Sciences). Specificity of the anti-VCBP-A/B and -C antibody has been previously investigated in depth [2]. The absence of cross reactivity between the anti-VCBP-A antibody and VCBP-B epitopes has been examined by SDS-PAGE and Western blot analysis performed on stomach and blood serum, in which VCBP -B and -A protein are present, respectively (Fig. S4).

### Whole mount *in situ* hybridization and immunohistochemistry

*Ciona* embryos and juveniles at different developmental stages, and digestive tract specimens were fixed either for 90 min at RT or overnight at 4°C in a mixture containing 4% paraformaldehyde, 0.1 M MOPS pH 7.5, 0.5 M NaCl. Samples were dehydrated in a series of ethanol solutions (30%, 50%, 70%) and stored at -20°C. WISH on larval and juvenile stages has been described previously [19], [20]. DIG-labelled riboprobes were used at a final concentration of 0.3 ng/µl for embryos and 0.1 ng/µl for juvenile stages and gastrointestinal tissue fragments, and incubated overnight at 55°C and 48°C, respectively. Nitroblue tetrazolium (NBT) and -bromo-4-chloro-3-indolyl-phosphate (BCIP) (Roche) were used for the signal detection.

Double fluorescent ISH was performed as described for WISH technique until the incubation step with the antibody. Before adding the horseradish peroxidase (POD)-conjugated anti-DIG antibody (Roche), samples were equilibrated in TNT (100 mM Tris pH 7.5, 150 mM NaCl, 0.1% Tween 20) and processed as described by Dufour and collaborators (2006) [21]. The TSA™-Plus Cyanine 3/Cyanine 5 System (Perkin Elmer) has been used for the detection of the labelled riboprobes.

WIHC was performed on BLR stage and stage 7 of 1^st^ ascidian juvenile. After rehydration, samples were washed in PBS pH 7.4, 2 for 5 min, and incubated in PBS pH 5.5 containing 0.5 mg/ml cellulase, for 10 min at 37°C, and then placed on ice for 10 min. Samples were washed in PBS pH 7.4, 0.25% Triton, 0.1% Tween 20, twice for 10 min at RT and incubated in 0.3% H_2_O_2_ in PBS pH 7.4. After two washes in PBS pH 7.4 for 10 min, the samples were incubated first in PBS pH 7.4, 0.25% Triton, 0.1% Tween 20, 5% normal goat serum for 2 hr at RT, and then with either anti-VCBP-A (2.12 µg/ml) or anti-VCBP-C (5.2 µg/ml) antibody, diluted in PBS pH 7.4, 5% normal goat serum, overnight at 4°C. After incubation, the specimens were washed in PBS pH 7.4, 0.2% Tween 20, 4 times for 15 min and twice for 30 min. The samples were equilibrated in PBS pH 7.4, 0.2% Tween 20, 1% BSA for 2 hr and then incubated overnight at 4°C with the biotinylated anti-rabbit IgG from the Vectastain Elite ABC Kit for peroxidase (Vector Laboratories). From this step the samples were processed according to manufacturer's recommendations. Detection used the Fast DAB set (Sigma Aldrich), according to manufacturer's recommendations. Controls were run in parallel by using preimmune rabbit IgG at the same concentration.

WISH and WIHC samples were observed under an Axiophot light microscope (Zeiss) equipped with Nomarsky optics, and images were acquired by Axiovision software. In some instances, hybridized samples were embedded in Epon 812 resin and semi-thin serial sections were cut and examined with phase-contrast optics. Double fluorescent ISH samples were observed under a confocal microscope LSM 510 META (Zeiss) and images were acquired by Zeiss LSM Image Browser.

### ISH on juvenile stomach sections and IHC on adult stomach sections

For ISH of 1–2 cm juveniles (small adults), samples were fixed in 4% paraformaldehyde in seawater overnight at 4°C. For IHC, dissected stomachs were fixed in Bouin's fluid (saturated picric acid: formaldehyde: acetic acid/15∶5∶1) for 24 hours. Samples were dehydrated and embedded in paraffin; 7 µm sections were cut with a microtome (RM2245, Leica) and collected onto Superfrost Plus slides (Thermo Scientific).

Tissue sections were processed for ISH as described previously [22] and hybridized overnight with 80 ng per slide of DIG labelled riboprobes at 55°C. NBT/BCIP substrates (Roche) were used for the signal detection. Parallel control experiments used the corresponding sense RNA probes.

Immunostaining of stomach sections was performed with the Vectastain Elite ABC Kit (Vector Laboratories) and the Fast DAB set (Sigma Aldrich). Sections were incubated with anti-VCBP-A (1.4 µg/ml) or anti-VCBP-A/B (4.9 µg/ml) or anti-VCBP-C (2.0 µg/ml) antibody diluted in TBS, 1% BSA, overnight at 4°C. Controls were run in parallel with preimmune rabbit IgGs, used at the same concentration of the anti-VCBPs antibodies.

### Immunogold on stomach sections

Stomach samples were fixed, overnight at 4°C, in a mixture of 0.5% glutaraldehyde and 2% paraformaldehyde in seawater. The following day, samples were thoroughly rinsed in seawater, dehydrated through a graded alcohol concentration series and then embedded in EPON resin and cut; 60 nm ultrathin sections were collected on 200 mesh nickel grids.

Sections were washed 3 times for 5 min in ddH_2_O, equilibrated for 15 min in TBS pH 8.8, containing 10% normal goat serum and washed in TBS pH 8.8, 3 times for 5 min. Then, grids were incubated with the anti-VCBP-A antibody (14 µg/ml) diluted in TBS pH 8.8 containing 1% BSA, overnight at 4°C in a humidified chamber. After incubation, grids were washed in TBS pH 8.8, 5 times for 2 min and then incubated, for 1 hr at RT, in TBS pH 8.8 containing biotinylated anti-rabbit IgG (1∶50) from the Vectastain Elite ABC Kit (Vector Laboratories). Sections were washed 5 times for 2 min with TBS pH 8.8, 0.05% Tween 20 and then incubated with streptavidin labelled with 20 nm gold particles (BBI International), diluted 1∶20 in TBS pH 8.8, for 1 hr. The grids were then washed in TBS pH 8.8, 0.05% Tween 20, 5 times for 2 min and finally in ddH_2_O, 3 for 2 min. Control experiments were run in parallel using preimmune rabbit IgG as primary antibody, at the same concentration of the anti-VCBP-A antibody. Sections were counterstained with lead citrate (30 sec) and observed using a Leo 912 (Zeiss) transmission electron microscope.

### Treatment with bacteria strains

*Bacillus cereus* (ATCC 1178) and *Escherichia coli* (ATCC 25922) were grown in LB Broth at 37°C overnight under orbital shaking. Bacteria were centrifuged at 3,000 rpm for 10 min at 4°C and cell pellets were resuspended in seawater. After inactivation, achieved through incubation at 60°C for 1 hr, bacteria were again centrifuged at 3,000 rpm for 10 min at 4°C, and cell pellets were resuspended in seawater.

*B. cereus* concentration was determined by counting the cell number in a Neubauer hemocytometer. *E. coli* concentration was determined by measuring the bacteria cultures OD_600 nm_ and assuming that 1.0 OD_600 nm_ = 8×10^8^ cell/ml.

*Ciona* juveniles, raised until the stage 7/8, were maintained in filtered seawater until the digestive tract was observed to be empty (∼2 days). The animals, developed from a single batch of gametes, were detached from the Petri dishes, split into two samples, transferred to 3 cm Petri dishes containing filtered seawater, and used as treated and control samples, respectively. Bacteria, either *B. cereus* or *E. coli*, were added to a final concentration of 10^6^ cells/ml. After 3 hr incubation with mild rotatory shaking, juveniles were collected by low speed centrifugation and used for RNA extraction.

### Quantitative real-time PCR

Oligo(dT)-primed single-stranded cDNA was synthesized from 1 µg of total RNA of embryo stages and the stage 7–8 of ascidian juvenile. Specific primers for *VCBP-A* (sense primer, 5′-ATTTGATACAGTGACGTGG-3′; antisense primer, 5′-CGGTAGTGGGAATATGAA-3′), *-B* (sense primer, 5′-GATCGAATGTTCTTACACTGG-3′; antisense primer, 5′-CCGCACTGATAATGAGAGA-3′), and *-C* (sense primer, 5′-AGACCAACGCCAACACAGTA-3′; antisense primer, 5′-CCCATACATTGCAGCATTTC-3′) were designed in conserved regions of the VCBP sequences identified in the numerous available *Ciona* EST libraries of embryo stages and adult tissues, registered in GenBank. Cytoskeletal actin (GenBank ID: AJ297725) was used as a reference gene. Actin-specific primers were: sense primer, 5′-CCCAAATCATGTTCGAAACC-3′; antisense primer, 5′-ACACCATCACCACTGTCGAA-3′.

The amplification efficiency of each primer set was assessed using serially 10-fold diluted cDNA.

RT- qPCR was performed on Vii™ 7 Real-Time PCR System (Life Technologies). FastStart SYBR Green Master (Roche) was used according to the manufacturer's protocol with a primer concentration of 0.28 nM/µl and 1 µl of cDNA (diluted 1∶10). Reaction conditions were as follows: denaturation at 95°C for 10 min and 40 amplification cycles (95°C for 15 sec and 60°C for 1 min).

Reactions were performed in triplicate on three different samples. Data were analyzed with Vii™ 7 Real-Time PCR software (Life Technologies), and quantified with the comparative Ct method (2^−ΔΔ*Ct*^) [23], based on Ct values for VCBP genes and the reference gene in order to estimate the relative levels of mRNA expression.

The significance of the relative ΔΔ*Ct*s of each group, compared to the controls, either unfertilized eggs or untreated stage 7–8 of ascidian juvenile, respectively, was determined using ‘independent samples *t*-test’. The statistical analyses were performed using SPSS software.

## Supporting Information

Figure S1 **Schematic representation of** ***VCBP-A*** **,** ***-B*** **, and** ***-C*** **expression from larva stage to “small adult”.**
(TIF)

Figure S2 **ISH carried out with** ***VCBP-A*** **,** ***-B*** **and** ***-C*** **sense probes.** No transcripts were detected in the control specimens from larva to “small adult” stages. (Scale bars 50 µm).
(TIF)

Figure S3 **WIHC performed with anti-VCBP-A and -C pre-immune sera.** No protein expression was detected in samples of BLR stage and stage 7 of 1^st^ ascidian juvenile. (Scale bars 50 µm).
(TIF)

Figure S4 **Reactivity of anti-VCBP-A/B and anti-VCBP-A antibodies with** ***Ciona intestinalis*** **stomach and blood serum proteins.** SDS-PAGE and Western blot analysis were performed as described by Dishaw et al. (2011) [1] on samples containing (A, C) 100 µg stomach proteins and (B, D) 7.5 µg blood serum proteins, prepared according to previously described protocols [1], [2]. Blotted proteins were probed with (A, B) the anti-VCBP-A/B and (C, D) anti-VCBP-A antibody, followed by HRP-conjugated secondary antibody and ECL-Plus detection. The anti-VCBP-A/B antibody detects (A) an ∼50 kDa and (B) an ∼35 kDa band in the stomach extract and blood serum, corresponding to VCBP-B and -A, respectively. The anti-VCBP-A antibody (C) does not react with any band of the stomach extract and (D) in blood serum, detects a single band corresponding to VCBP-A protein. On the left, relative mobilities of molecular weight markers (Healthcare, Life Sciences) are shown.
(DOC)
