# Supplementary figures and images for: Expression of Ciona intestinalis Variable Region-Containing Chitin-Binding Proteins during Development of the Gastrointestinal Tract and Their Role in Host-Microbe Interactions
Source: PLoS One. 2014 May 2;9(5):e94984. doi: 10.1371/journal.pone.0094984 (PMC4008424; doi:10.1371/journal.pone.0094984)

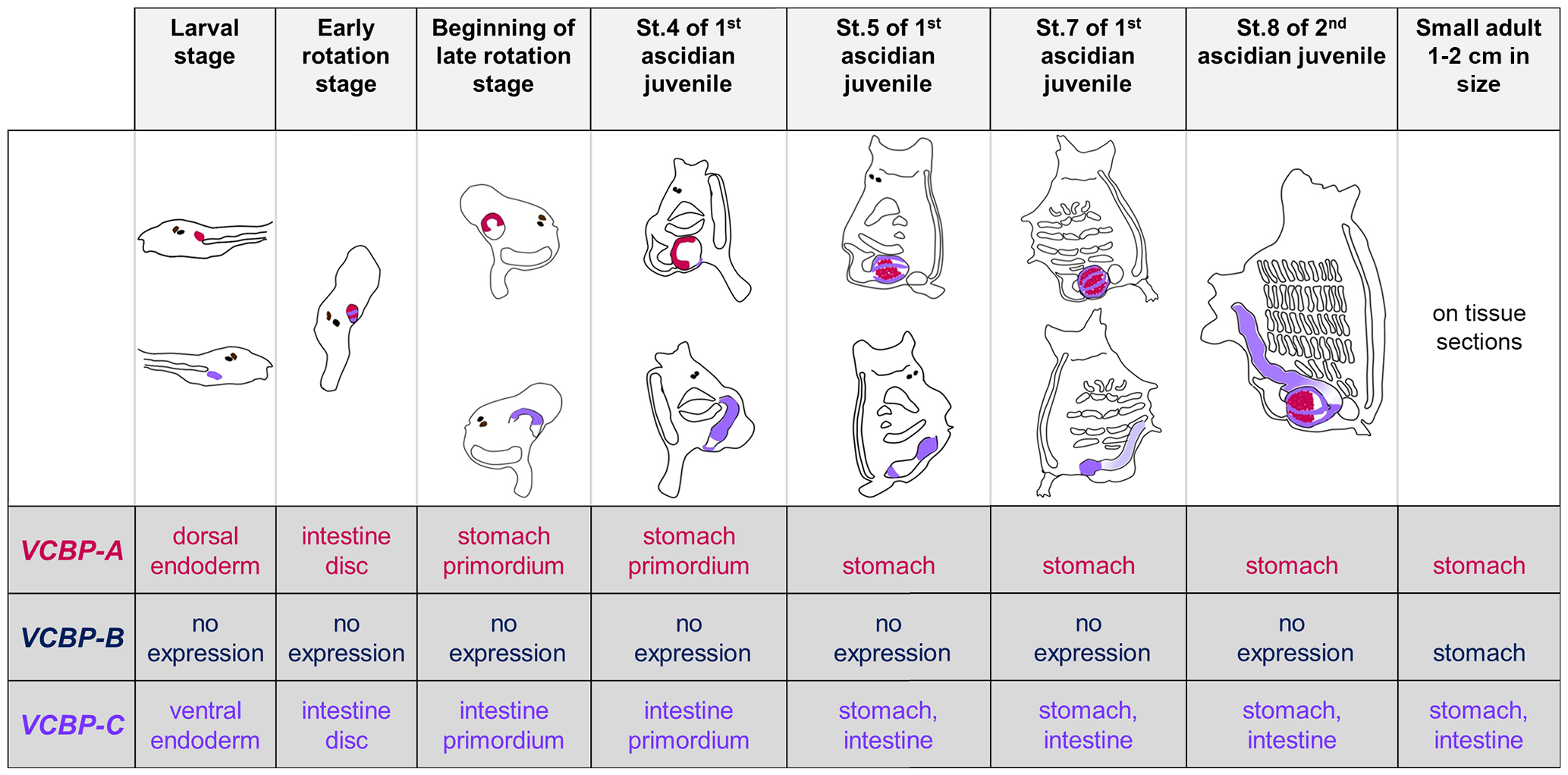

Supplement: Figure S1 — Schematic representation of VCBP-A , -B , and -C expression from larva stage to “small adult”. (TIF) [file pone.0094984.s001.tif]

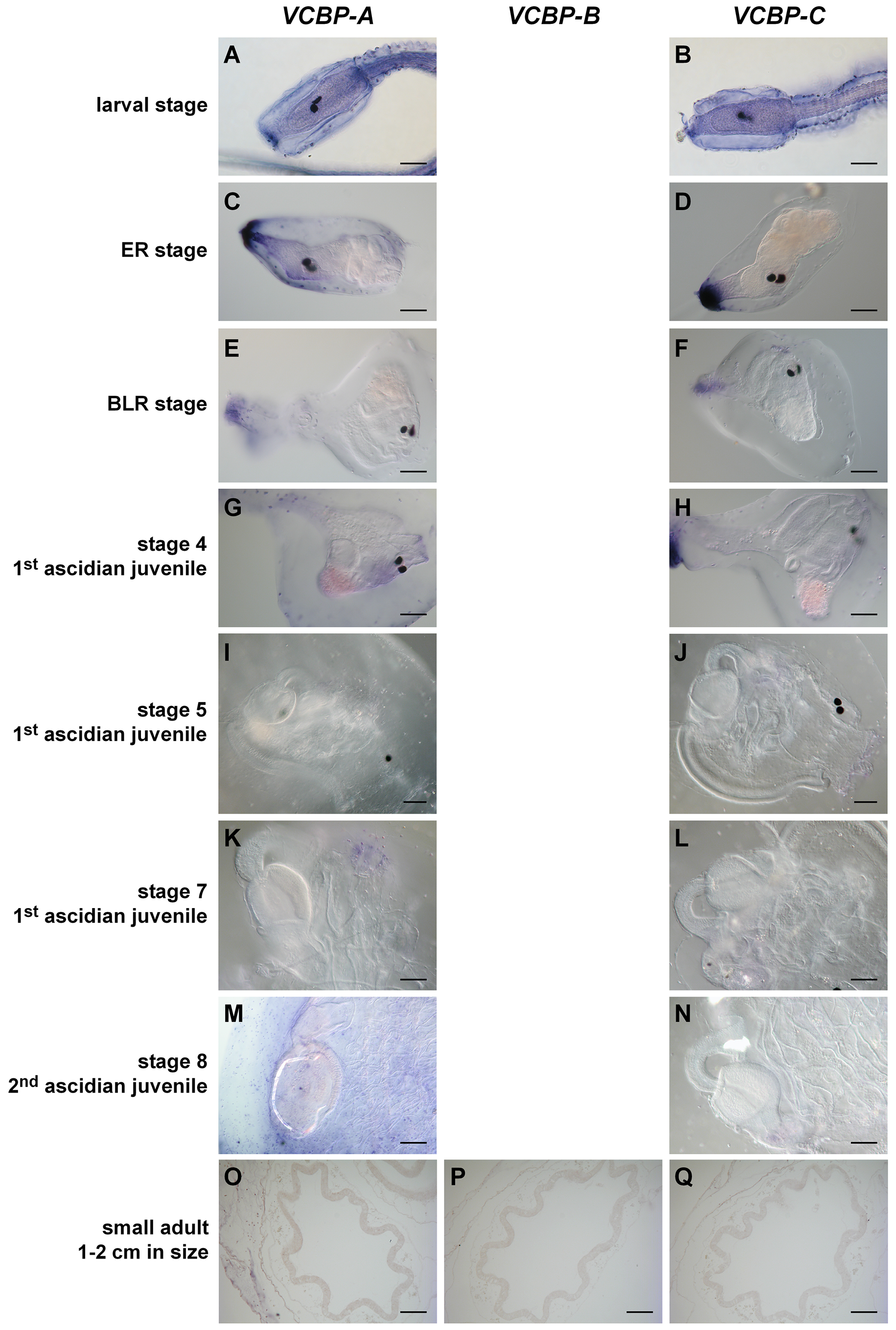

Supplement: Figure S2 — ISH carried out with VCBP-A , -B and -C sense probes. No transcripts were detected in the control specimens from larva to “small adult” stages. (Scale bars 50 µm). (TIF) [file pone.0094984.s002.tif]

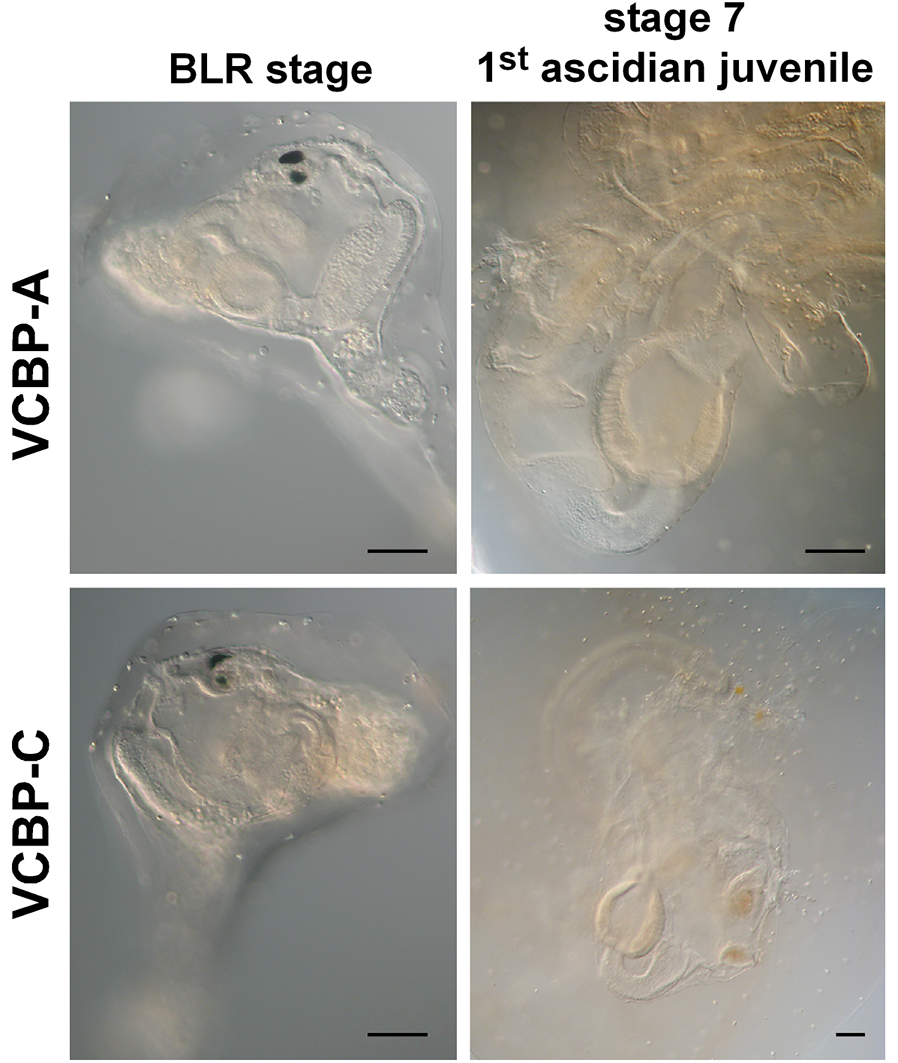

Supplement: Figure S3 — WIHC performed with anti-VCBP-A and -C pre-immune sera. No protein expression was detected in samples of BLR stage and stage 7 of 1st ascidian juvenile. (Scale bars 50 µm). (TIF) [file pone.0094984.s003.tif]
