# Supplementary material for: Expression of Ciona intestinalis Variable Region-Containing Chitin-Binding Proteins during Development of the Gastrointestinal Tract and Their Role in Host-Microbe Interactions
Source: PLoS One. 2014 May 2;9(5):e94984. doi: 10.1371/journal.pone.0094984 (PMC4008424; doi:10.1371/journal.pone.0094984)
Supplement: Figure S4 — Reactivity of anti-VCBP-A/B and anti-VCBP-A antibodies with Ciona intestinalis stomach and blood serum proteins. SDS-PAGE and Western blot analysis were performed as described by Dishaw et al. (2011) [1] on samples containing (A, C) 100 µg stomach proteins and (B, D) 7.5 µg blood serum proteins, prepared according to previously described protocols [1], [2]. Blotted proteins were probed with (A, B) the anti-VCBP-A/B and (C, D) anti-VCBP-A antibody, followed by HRP-conjugated secondary antibody and ECL-Plus detection. The anti-VCBP-A/B antibody detects (A) an ∼50 kDa and (B) an ∼35 kDa band in the stomach extract and blood serum, corresponding to VCBP-B and -A, respectively. The anti-VCBP-A antibody (C) does not react with any band of the stomach extract and (D) in blood serum, detects a single band corresponding to VCBP-A protein. On the left, relative mobilities of molecular weight markers (Healthcare, Life Sciences) are shown. (DOC) [file pone.0094984.s004.doc]

**
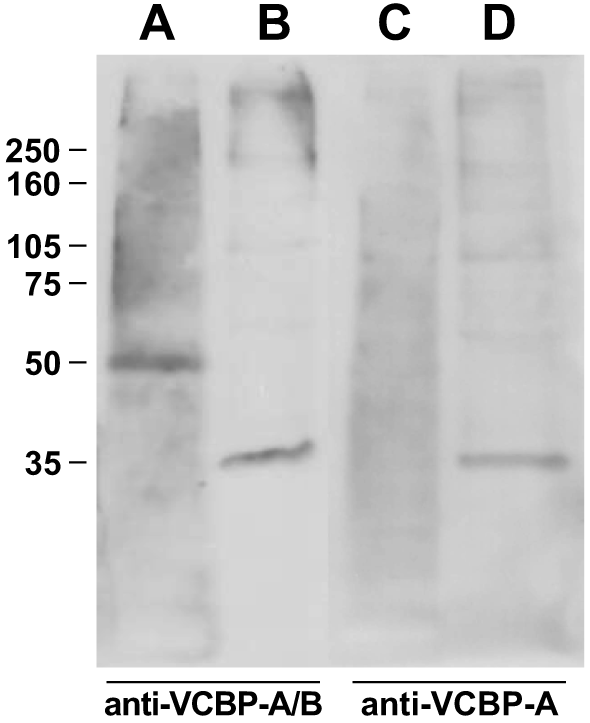
**

**References**

1. Dishaw LJ, Giacomelli S, Melillo D, Zucchetti I, Haire RN, et al. (2011) A role for variable region-containing chitin-binding proteins (VCBPs) in host gut-bacteria interactions. Proc Natl Acad Sci U S A 108: 16747-16752.
2. Pinto MR, Chinnici CM, Kimura Y, Melillo D, Marino R, et al. (2003) CiC3-1a-mediated chemotaxis in the deuterostome invertebrate *Ciona intestinalis* (Urochordata). J Immunol 171: 5521-5528.
